# Truncation-Driven Lateral Association of α-Synuclein Hinders Amyloid Clearance by the Hsp70-Based Disaggregase

**DOI:** 10.3390/ijms222312983

**Published:** 2021-11-30

**Authors:** Aitor Franco, Jorge Cuéllar, José Ángel Fernández-Higuero, Igor de la Arada, Natalia Orozco, José M. Valpuesta, Adelina Prado, Arturo Muga

**Affiliations:** 1 Department of Biochemistry and Molecular Biology, Faculty of Science and Technology, University of the Basque Country (UPV/EHU), 48080 Bilbao, Spain; aitor.francob@ehu.eus (A.F.); joseangel.fernandez@ehu.eus (J.Á.F.-H.); adelina.prado@ehu.eus (A.P.); 2 Instituto Biofisika (UPV/EHU, CSIC), University of the Basque Country, 48940 Leioa, Spain; igor.delaarada@ehu.eus (I.d.l.A.); nataliaorozko@hotmail.com (N.O.); 3 Department of Macromolecular Structure, Centro Nacional de Biotecnología (CNB-CSIC), 28049 Madrid, Spain; jcuellar@cnb.csic.es (J.C.); jmv@cnb.csic.es (J.M.V.); 4 Fundación Biofísica Bizkaia/Biofisika Bizkaia Fundazioa (FBB), Barrio Sarriena s/n, 48940 Leioa, Spain

**Keywords:** α-synuclein, amyloid disassembly, suprafibrillar assemblies, chaperone, human disaggregase, Hsp70, Hsp40

## Abstract

The aggregation of α-synuclein is the hallmark of a collective of neurodegenerative disorders known as synucleinopathies. The tendency to aggregate of this protein, the toxicity of its aggregation intermediates and the ability of the cellular protein quality control system to clear these intermediates seems to be regulated, among other factors, by post-translational modifications (PTMs). Among these modifications, we consider herein proteolysis at both the N- and C-terminal regions of α-synuclein as a factor that could modulate disassembly of toxic amyloids by the human disaggregase, a combination of the chaperones Hsc70, DnaJB1 and Apg2. We find that, in contrast to aggregates of the protein lacking the N-terminus, which can be solubilized as efficiently as those of the WT protein, the deletion of the C-terminal domain, either in a recombinant context or as a consequence of calpain treatment, impaired Hsc70-mediated amyloid disassembly. Progressive removal of the negative charges at the C-terminal region induces lateral association of fibrils and type B* oligomers, precluding chaperone action. We propose that truncation-driven aggregate clumping impairs the mechanical action of chaperones, which includes fast protofilament unzipping coupled to depolymerization. Inhibition of the chaperone-mediated clearance of C-truncated species could explain their exacerbated toxicity and higher propensity to deposit found in vivo.

## 1. Introduction

**Figure 1:**
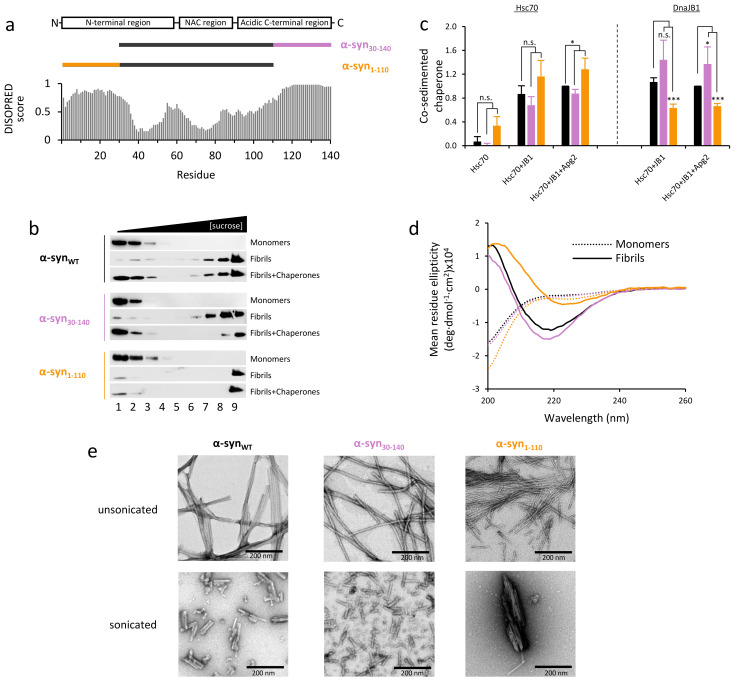
Truncation of the last 30 residues of α-syn induces fibril lateral association and blocks fibril disaggregation by chaperones. (**a**) Full-length α-syn and truncation mutant sequence scheme laid out on a disorder prediction obtained with DISOPRED3 software (0 = ordered, 1 = disordered). (**b**) Fibril disaggregation of full-length and truncated mutants of α-syn analyzed by sucrose-gradient fractionation. Reactions of 10 µM α-syn monomers, sonicated fibrils or sonicated fibrils, incubated with 10 µM Hsc70, 5 µM DnaJB1 and 1 µM Apg2 for 2.5 h, were loaded on a 5–40% sucrose-gradient and ultracentrifuged. Fractions were analyzed by SDS-PAGE and immunoblotting. (**c**) The relative amount of Hsc70 (left panel) and DnaJB1 (right panel) bound to fibrils for different chaperone mixtures was quantified using a co-sedimentation assay of 2 μM α-syn fibrils (full-length or truncation mutants) and Hsc70 (2 μM) in the presence of DnaJB1 (1 μM) alone or with Apg2 (0.2 μM). As controls, the same chaperone mixtures were analyzed in the absence of fibrils, and these signals were subtracted. Data were normalized against α-syn_WT_ in the presence of the three chaperones (n.s.: not significant; * *p* < 0.05; ** *p* < 0.01; *** *p* < 0.001). (**d**) The secondary structure of monomeric and fibrillar species of α-syn_WT_ and truncated mutants was compared by Far-UV CD (**d**) and their morphology before and after sonication was evaluated by EM (**e**). Color code: α-syn_WT_ (black), α-syn_30-140_ (violet) and α-syn_1-110_ (orange).

α-Synuclein (α-syn) is a presynaptic protein whose biological function remains unclear, although it has been implicated in diverse physiological processes including the regulation of synaptic transmission, calcium regulation or mitochondrial homeostasis [1]. It represents approximately 1% of the total cytosolic protein in the central nervous system [2] and can be found either soluble, adopting a largely disordered conformation, or membrane-associated, partially folding into an amphipathic α-helix. This 140-amino-acid-long protein contains three regions (Figure 1a): an amphipathic N-terminal region comprising residues 1–60; a non-amyloid β component (NAC) region (residues 61–95) characterized by the presence of hydrophobic residues; a C-terminal region (residues 96–140) enriched in acidic residues and responsible for its overall negative charge. Mutations on the α-syn gene (*SNCA*) or age-related decline of the protein quality control system [3] favor α-syn aggregation into pathological amyloid fibrils. The deposition of α-syn amyloid aggregates into the so-called Lewy bodies (LBs) and Lewy neurites (LNs) is a hallmark in patients with Parkinson’s disease and other synucleinopathies such as dementia with Lewy bodies and multiple system atrophy [4].

A plethora of post-translational modifications (PTMs), including phosphorylation, ubiquitination, nitration, O-GlcNAcylation and truncation, seem to play a key role in α-syn pathogenesis [5]. Truncation is one of the predominant modifications of α-syn. Both N- and C-terminally truncated α-syn species have been found in the brains of healthy and diseased individuals [6,7,8,9]. Epitope mapping and mass spectroscopy (MS) have confirmed the presence of truncated α-syn species starting at residues 5, 10, 18, 19 and 68 (N-truncations) and others cleaved after residues 103, 110, 113, 114, 115, 119, 122, 124, 125, 133 and 135 (C-truncations) [10]. Interestingly, many of these truncations are enriched within the insoluble fraction of diseased brain lysates compared to healthy controls, pointing to the important role that truncation could play in pathogenesis [7,8,11,12].

In the intracellular medium, proteins confront proteases that partially or fully degrade them. In the case of α-syn, distinct proteases can process differently its various assembly states (monomeric, oligomeric or fibrillar), generating several truncated forms. Among lysosomal proteases, cathepsin D (CstD) is able to C-truncate both monomeric and fibrillar α-syn; cathepsin B (CtsB) degrades monomers but only truncates fibrils, while cathepsin L (CtsL) can fully degrade all forms of α-syn [10]. The cytosolic protease calpain-1 cleaves monomeric α-syn at its N-terminus and NAC region and fibrillized α-syn predominantly at the C-terminus [13]. Monomeric α-syn is also processed by the 20S proteasome and caspase I, both cleaving at the protein C-terminus [14,15,16]. Other proteases that contribute to α-syn truncation include the lysosomal protease asparagine endopeptidase (AEP) and extracellular proteases such as neurosin/kallikrein-6, matrix metalloproteases 1 and 3 (MMP1 and MMP3) and plasmin [10]. The combined action of these proteases results in the formation of numerous truncated forms at different stages of the aggregation process.

Although it has been shown that truncation strongly influences α-syn aggregation and prion-like pathogenicity [14,17,18], very little is known about the effect of this PTM on aggregate clearance. α-Syn fibrils and oligomers are disassembled by the human disaggregase, a chaperone system composed of three proteins: Hsc70 (a constitutively expressed member of the Hsp70 family), DnaJB1 (a representative of the class B Hsp40 or J-protein family) and Apg2 (a nucleotide exchange factor of the Hsp110 family) [19,20]. Fibrils of a truncated mutant of α-syn lacking the N- and C-terminus (α-syn_30–110_) are not disassembled by this machinery, a finding that was explained assuming that the N- and/or C-terminal parts of the α-syn molecule might provide a specific binding platform for the chaperones [19]. It has been recently shown that DnaJB1 mainly interacts with the C-terminal region of α-syn fibrils, whereas Hsc70 can bind to the N-terminal and NAC regions [21]. Truncation of the last 30 residues of α-syn also abrogates fibril solubilization, as it avoids the first and limiting step of the reaction, e.g., the initial binding of the cochaperone DnaJB1 to the fibril surface necessary to recruit Hsc70, whereas deletion of the first 29 residues only reduces slightly the disaggregase activity [21]. We analyze herein the consequences that the deletion of different C-terminal regions of α-syn has on the conformation and macroscopic properties of α-synuclein aggregates and relate them to the interaction with different combinations of the human disaggregase components and the ability of the Hsc70-based machinery to disassemble fibrils and oligomers of α-syn. We discuss the putative implications that our data might have in aggregate clearance and thus in pathogenesis.

## 2. Results

### 2.1. Effect of α-Syn N- and C-Terminal Truncation on Chaperone Activity and Interaction

The electron microscopy (EM) structure of recombinantly produced α-syn fibrils showed that both the N- and C-terminal ends of the protein are not visible, indicating a high degree of flexibility due to their predicted intrinsically disordered structure (Figure 1a) [22,23,24,25]. NMR data and limited proteolysis proved these terminal residues to be solvent-exposed and unprotected from protease attack [26]. To understand the importance of these unstructured regions in chaperone-mediated fibril disaggregation, we generated two mutants lacking either the first 29 N-terminal residues (α-syn_30-140_) or the last 30 C-terminal amino acids (α-syn_1–110_). After one week of aggregation, amyloid fibrils of α-syn_WT_ and both mutants were subjected to sonication, as fibril fragmentation facilitates an efficient disassembly by the disaggregase [20]. Sonicated fibrils were incubated with the chaperone mixture in the presence of ATP and an ATP regeneration system, and the reaction products were fractionated in a density-gradient centrifugation and analyzed by immunoblotting (Figure 1b). Considering their molecular mass, monomers are expected at the top of the gradient, oligomers at intermediate positions and aggregates at the bottom. After incubation with chaperones, α-syn_WT_ and α-syn_30-140_ were detected in fractions 1–3, confirming their disassembly into soluble monomers. Conversely, α-syn_1-110_ was mainly detected in fraction nine, indicating that this truncation mutant was not susceptible to disaggregation, in good agreement with recent observations [21].

Disaggregation of fibrils requires binding of the three chaperone components to the aggregates [19]. Both N- and C-terminal residues have been proven important for the interaction of different heat shock proteins with monomeric α-syn [21,27,28]. Hsc70 binds α-syn preferentially through its N-terminus [21,28], while DnaJB1 binds to its C-terminal domain [21,27]. As DnaJB1 is the first chaperone that binds to fibrils, recruiting Hsc70 to the aggregate surface [19], we first thought that the interaction of DnaJB1 with α-syn_1-110_ could be impaired, as suggested in a recent study [21]. To verify if this was the case, we performed two types of experiments. In the first one, the ability of each chaperone to prevent aggregation of α-syn_1-110_ was assayed. To this aim, we followed the increase in ThT fluorescence of self-seeded α-syn_1-110_ in the absence and presence of each of the three components of the human disaggregase system at a 10:1 (α-syn/chaperone) molar ratio. Aggregation was delayed in the presence of any of the chaperones (Figure S1a), indicating that the three chaperones bind C-truncated α-syn. Our data do not allow identifying the α-syn species that interact with the chaperones, as they can use different mechanisms to prevent aggregation, including inhibition of primary nucleation via monomer interaction and elongation/secondary nucleation blockade through seed neutralization [29]. We then sought to determine if the DnaJB1-mediated recruitment of Hsc70 to α-syn_1-110_ fibrils was compromised. Through a co-sedimentation assay, we saw that Hsc70 bound poorly to WT fibrils in the absence of DnaJB1 and that cochaperone addition increased 12-fold the amount of fibril-bound chaperone (Figure 1c and Figure S1b), which is maintained in the presence of Apg2, following a comparable association pattern seen for amorphous aggregates [30]. A similar Hsc70 recruitment in the absence and presence of cochaperones was observed for the α-syn variants lacking either of the terminal regions. These data indicate that disaggregation of α-syn_1-110_ fibrils by chaperones is blocked, although their interaction with the aggregated substrate is not impaired. They also revealed that the amount of DnaJB1 bound to α-syn_1-110_ fibrils is reduced 40–50% compared to α-syn_WT_ and α-syn_30-140_ (Figure 1c), thus suggesting that the remaining bound cochaperone efficiently transfers Hsc70 molecules to the aggregate surface so that the aggregate-bound chaperone estimated for fibrils of WT and the two α-syn deletion mutants is similar under these experimental conditions, e.g., chaperone excess. This interpretation is consistent with the finding that one substrate-bound Hsp40 dimer can recruit several Hsp70 molecules to the aggregate surface [21,30,31].

### 2.2. α-Syn Fibrils Lacking the C-Terminal Residues Render More Stable Structures Due to Lateral Association 

The formation of a different aggregate structure due to C-terminal truncation could explain the difficulty of chaperones to disaggregate α-syn_1-110_ fibrils. Indeed, it has been recently described that a C-terminal truncated mutant of α-syn (α-syn_1-108_) forms amyloid fibrils with a distinct structure and morphology [32]. In this deletion mutant, the canonical circular dichroism (CD) spectrum of amyloid fibrils with a broad minimum at 218 nm and a positive maximum at 200 nm was red-shifted, a behavior we replicate with the α-syn_1-110_ mutant and not with α-syn_30-140_ (Figure 1d). This red-shift has been associated with strongly twisted β-sheets [32], in good agreement with recent cryo-EM structural data indicating that the fibril helical twist increased upon removal of C-terminal residues [25]. EM analysis of these samples showed that, unlike α-syn_WT_ and α-syn_30-140_, which formed fibrils with rod-like morphologies and a limited clumping, α-syn_1-110_ fibrils associated laterally and clumped together into more stable (Figure S1c) suprafibrillar morphologies (Figure 1e), a behavior described for a similar mutant α-syn_1-108_ [32,33]. After sonication, the three variants fragmented into shorter fibrils, but only fragments of the α-syn_1-110_ mutant clumped together into large structures (Figure 1e and Figure S1d). This behavior suggests that disaggregation failure could be due to the lateral association of fibrils into these large and stable structures.

**Figure 2:**
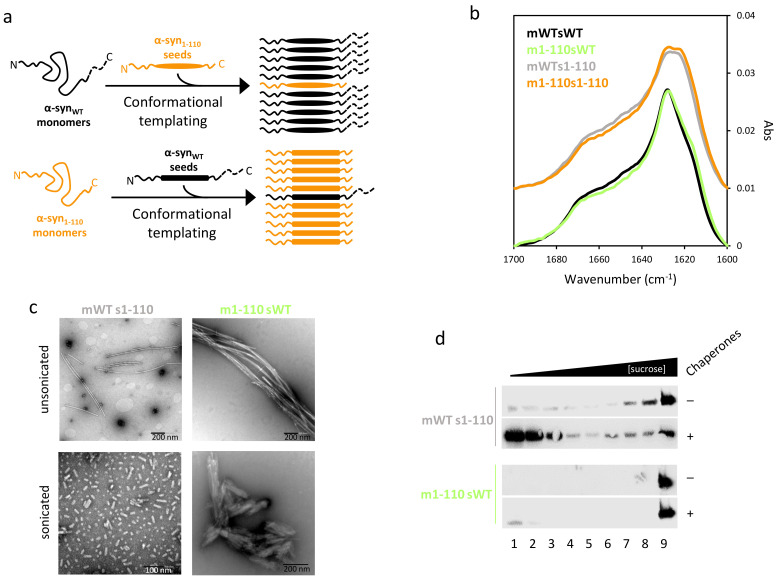
Stable suprafibrillar assemblies of cross-seeded fibrils of C-terminal truncated α-syn species challenges the human disaggregase. Fibrils produced by incubation of monomeric α-syn_WT_ with α-syn_WT_ (mWTsWT) or α-syn_1-110_ (mWT s1-110) seeds, and monomeric α-syn_1-110_ with α-syn_1-110_ (m1-110s1-110) or α-syn_WT_ (m1-110sWT) seeds, (**a**) were analyzed by FT-IR spectroscopy (**b**). Cross-seeded unsonicated and sonicated samples were then analyzed by EM (**c**). Sonicated cross-seeded fibrils were mixed with the human disaggregase, and their disassembly susceptibility was followed by sucrose-gradient separation (**d**).

To explore the factors that regulate the formation of suprafibrillar assemblies of α-syn_1-110_, we performed cross-seeding experiments with α-syn_WT_ and α-syn_1-110_ (Figure 2a). We aggregated monomeric α-syn_WT_ with α-syn_WT_ or α-syn_1-110_ seeds (mWTsWT and mWTs1-110, respectively) and monomeric α-syn_1-110_ with α-syn_WT_ or α-syn_1-110_ seeds (m1-110sWT and m1-110s1-110, respectively). The resulting fibrils were analyzed by FT-IR spectroscopy (Figure 2b) since, as compared with CD, it has a higher sensitivity to detect β-sheets in aggregated proteins and it does not suffer from differential absorption flattening effects due to the presence of aggregates [34,35]. Self-seeded samples showed spectra similar to those obtained for non-seeded α-syn_WT_ and α-syn_1-110_ (Figure S2a), with the strongest amide I band components between 1635 and 1610 cm^−1^, as expected from a conformation with a high content of β-structure. The spectrum of m1-110s1-110, but not mWTsWT, also showed broadening of the low-frequency amide I band between 1630 and 1610 cm^−1^, as found for a shorter α-syn variant [32]. The comparison of the spectra of cross-seeded samples revealed that seeds imposed their structural characteristics, a behavior widely established and termed conformational templating [36]. Then, we explored the behavior of cross-seeded samples upon sonication by EM (Figure 2c). mWTs1-110 fibrils, which resemble α-syn_1-110_ in terms of secondary structure, showed a reduction in size after sonication, whereas m1-110sWT, with a WT-like secondary structure, did not, forming instead suprafibrillar structures similar to pure α-syn_1-110_ fibrils. Sonicated cross-seeded fibrils were then incubated with chaperones to test their susceptibility to disaggregation (Figure 2d). mWTs1-110 fibrils were disaggregated, yielding soluble monomeric α-syn at the top of the density gradient, while m1-110sWT fibrils were not susceptible to disaggregation. Altogether, our data indicate that the lateral association of fibrils is not determined by the secondary structure that the protein adopts within the fibril, but rather depends on the absence of the last 30 residues. In addition, formation of such assemblies correlates with a reduced chaperone disaggregase activity.

### 2.3. The Disaggregase Activity of Chaperones Is Sensitive to the Length of the Region Deleted at C-Terminal of α-syn

**Figure 3:**
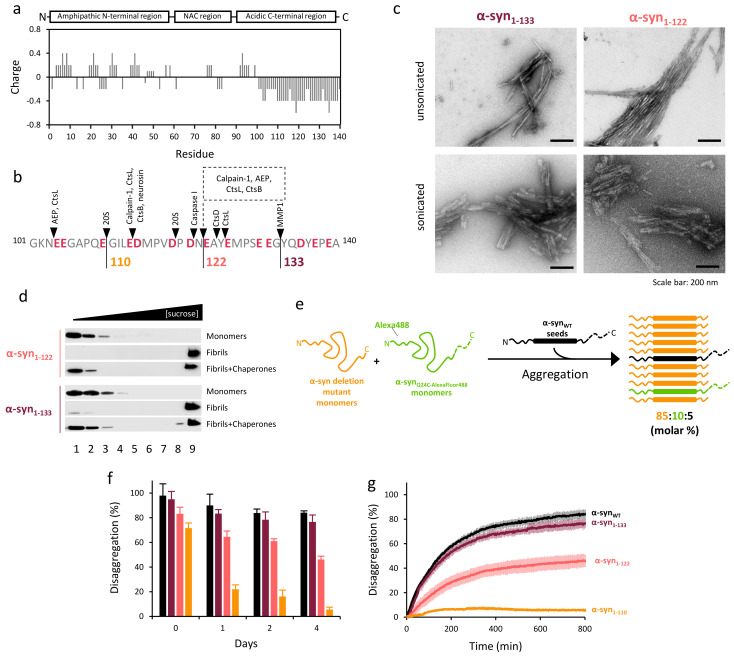
Chaperone-mediated disaggregation is gradually reduced as truncation of α-syn C-terminal domain increases. (**a**) Charge distribution of α-syn showing a concentration of negatively charged amino acids at its C-terminus. (**b**) Cleavage sites described for different proteases at the C-terminal region are marked with black arrows and negatively charged residues are highlighted in red. The three terminal truncation mutants analyzed in this work are marked and color-coded as in the next panels. (**c**) Unsonicated and sonicated α-syn_1-122_ and α-syn_1-133_ fibrils were analyzed by EM. (**d**) Sonicated samples were mixed with the human disaggregase to check their disassembly susceptibility. Disaggregation reactions were analyzed by sucrose-gradient fractionation followed by SDS-PAGE and immunoblotting of the fractions. (**e**) Fluorescently labeled fibril preparation scheme. (**f**) Disaggregation percentage of the different hybrid fibril preparations (2 µM) after 800 min incubation with chaperones at a α-syn/Hsc70 1:1 molar ratio. Chaperones were added the same day of fibril purification and sonication (day 0) or after incubating the fibrils at room temperature 1, 2 and 4 days. (**g**) Disaggregation kinetics of hybrid fibrils incubated for 4 days after sonication. Percentage of disaggregation in panels f and g was obtained as described in the Materials and Methods section.

The C-terminal domain of α-syn is a highly acidic region (Figure 3a) and under physiological conditions can be cleaved at different positions by several proteases (Figure 3b) [10]. In this context, we sought to determine if the length of the proteolyzed C-terminal region affected the solubilizing activity of the human disaggregase. To this aim, we compared the conformational properties and chaperone-mediated disaggregation of α-syn_WT_ and α-syn_1-110_ with those of two other physiologically relevant truncation mutants, α-syn_1-122_ and α-syn_1-133_. 

The differential FT-IR spectra (Figure S2b) obtained after subtracting the IR spectrum of each truncated mutant from that of the WT protein (Figure S2a) showed a progressive conversion from the α-syn_WT_ to the α-syn_1-110_ conformation as the length of the region deleted increased. The first conformational change occurred upon deletion of the last seven residues (α-syn_1-133_) and was evidenced by a slight broadening of the main amide I band component, which was better seen as a positive band at 1620 cm^−1^ in the difference spectrum. Further deletion of 18 and 30 residues strengthened broadening of the β-sheet spectral region, resulting in an increase in the intensity of the 1620 cm^−1^ band and the appearance of the following differential features: a positive band at around 1635 cm^−1^ and two negative bands at 1650 and 1674 cm^−1^. Therefore, these data suggest that the main structural rearrangement of α-syn starts upon the deletion of the last 18 residues at its C-terminal region and is compatible with an increase in the β-structure content (1620 and around 1633 cm^−1^), with the concomitant decrease in turns (1674 cm^−1^) and helical/unordered conformations (1650 cm^−1^) [34].

EM images of unsonicated α-syn_1-133_ and α-syn_1-122_ fibrils showed that both truncation mutants displayed increased lateral association compared to α-syn_WT_ (Figure 3c). Upon sonication, fibrils of both mutants were fragmented and tended to cluster together, forming clumps of fragmented fibrils similar but less densely packed than those of α-syn_1-110_. Sonicated fibrils of these two mutants were then incubated with chaperones, and disaggregation was followed by a sucrose-gradient fractionation (Figure 3d). Monomeric α-syn appeared at the top of the gradient, showing that fibrils of both mutants were susceptible to disaggregation. These data indicate that although the lateral association is increased for fibrils of these two partially truncated mutants, it may not be as strong as in the α-syn_1-110_ variant. 

The qualitative analysis of the disaggregase activity of the chaperone mixture by Western blot (WB) precluded a direct comparison of the different α-syn deletion variants. Therefore, we resorted to fluorescence dequenching measurements to compare the chaperone-induced disaggregation kinetics of these samples. To generate fluorescently labeled fibrils, monomers of each C-terminal truncation mutant were aggregated in the presence of AlexaFluor488 labeled α-syn_Q24C_ monomers and seeds of α-syn_WT_. The resulting hybrid fibrils contained 85:10:5 (molar percentage) of truncated mutant/α-syn_Q24C_-488/α-syn_WT_ seeds (Figure 3e) and might better mimic the in vivo situation in which different α-syn species are found in protein deposits [7,8,11,12]. Immediately after sonication (day 0), the different fibril preparations were mixed with the human disaggregase, and their disassembly was followed by fluorescence dequenching, as the fluorophore self-quenches in the highly packed fibrillar sample, even at low labeling efficiencies [20,37]. Compared to α-syn_WT_, we saw a gradual reduction of the disaggregation yield as the length of the truncated region at the C-terminus increased (Figure 3f). Intriguingly, hybrid fibrils that formed with the α-syn_1-110_ mutant showed a final disaggregation as high as 70%, contrasting with the results obtained for fibrils entirely composed of this mutant (Figure 1b). This may be explained by the presence of the entire C-terminal domain in 15% of the molecules that form the hybrid fibrils, which may be enough to weaken interfibril association through electrostatic repulsion. Interestingly, the difference in the disaggregation yield between fibrils containing α-syn_WT_ or truncated mutants increased with the incubation time at room temperature (Figure 3f). The efficiency of the human disaggregase to solubilize hybrid fibrils formed with α-syn_WT_ or α-syn_1-133_ showed, four days after sonication, a small (around 13%) reduction (Figure 3f,g). In contrast, the decrease observed for fibrils made of α-syn_1-122_ or α-syn_1-110_ mutants was significantly stronger. We hypothesize that, unlike α-syn_1-110_ pure fibrils, which rapidly cluster after sonication, the presence of a minor population (15%) of α-syn molecules with an intact C-terminal domain in the hybrid fibrils could slow down the fibril rearrangement that modulates chaperone-mediated disaggregation.

Summing up, we show that progressive deletion of the C-terminus results in a gradual decrease in the ability of chaperones to disaggregate α-syn fibrils. This behavior could be related to the reduction in the number of negative charges present in the C-terminus, which in turn promotes fibril lateral association [33,38,39].

### 2.4. Disaggregation of Calpain-Cleaved α-Syn Fibrils

**Figure 4:**
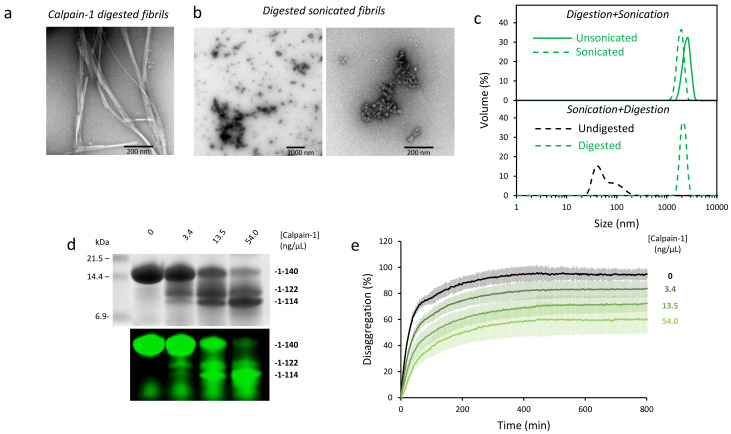
Cleavage of α-syn_WT_ fibrils with calpain-1 induces their lateral association and reduces chaperone-mediated disaggregation. EM images of α-syn unsonicated (**a**) and sonicated (**b**) fibrils digested with calpain-1 (13.50 ng/µL). (**c**) Size distribution measured by DLS of unsonicated and sonicated calpain-1 digested fibrils (upper panel) or undigested and digested sonicated fibrils (lower panel). (**d**) Sonicated α-syn fibrils were incubated at 37 °C for 10 min in the absence or presence of different calpain-1 concentrations. Then, after 2 h incubation with a calpain-1 inhibitor (SJA6017), samples were analyzed by SDS-PAGE. Gel was visualized by Coomassie blue staining (upper panel) or in-gel fluorescence of AF488-labeled α-syn in a VersadocMP. (**e**) Aliquots of the same samples (2 µM final concentration) were mixed with chaperones (1:0.5 α-syn/Hsc70 molar ratio) and their disaggregation kinetics were followed by means of fluorescence dequenching.

Physiological C-terminal truncation of α-syn renders heterogeneous aggregate populations, with varying amounts of uncleaved and cleaved protomers at different sites. In this context, we decided to cleave α-syn_WT_ fibrils in vitro with calpain-1. This cytosolic protease hydrolyzes monomeric α-syn at different sites located in the N-terminal and NAC regions but primarily cleaves fibrillized α-syn at the C-terminal domain [13]. We confirmed these results (Figure S3a), obtaining two main fragments after digestion of α-syn fibrils, previously identified as α-syn_1-122_ and α-syn_1-114_ [13]. Increasing concentrations of calpain-1 resulted in an enhanced generation of low molecular mass fragments. Interestingly, fibril digestion did not change significantly the secondary structure of the sample as seen by Far-UV CD (Figure S3b), suggesting that its structural integrity was maintained. EM (Figure 4a,b) and dynamic light scattering (DLS) (Figure 4c) showed that proteolysis triggered clumping of sonicated fibrils into larger particles, in contrast to what was seen for the full-length protein (α-syn_WT_; Figure 1e). 

Next, we checked the effect of calpain-1 cleavage on chaperone-mediated disaggregation. For that purpose, fluorescently labeled α-syn fibrils were sonicated and incubated with increasing amounts of calpain-1, rendering samples with a different proportion of full-length and truncated bands (Figure 4d). Importantly, in-gel fluorescence visualization showed that the two main fragments contained the AlexaFluor488 fluorophore, located in the N-terminal domain, confirming that cleavage occurred at the C-terminus. Chaperone-mediated disaggregation kinetics of the different samples was followed as a fluorescence dequenching process (Figure 4e). As the relative proportion of the C-terminal truncation fragments increased with calpain-1 concentration, the disassembly activity of the human disaggregase was reduced. Such a reduction confirmed the results obtained with recombinant truncation mutants and points towards physiological C-terminal truncation of α-syn as a key pathological event that could impair chaperone-mediated aggregate disassembly.

### 2.5. C-Terminal Truncation also Impairs Disaggregation of α-Syn Oligomers 

**Figure 5:**
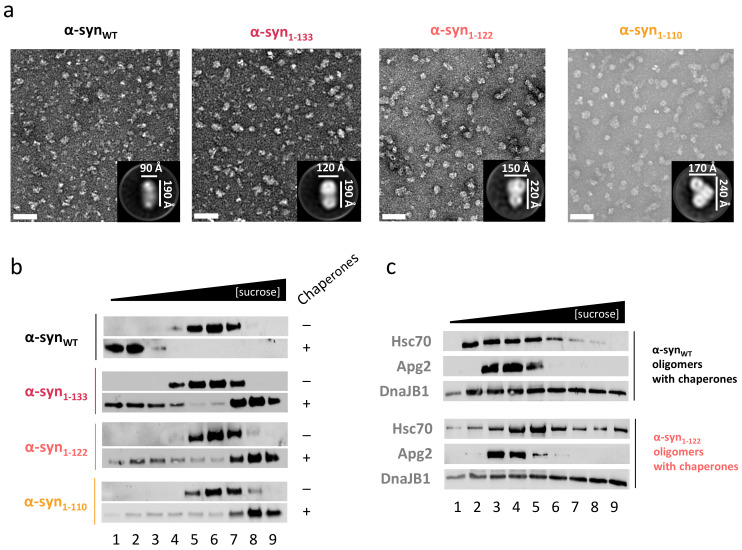
Effect of α-syn C-terminal truncation on the chaperone-induced disaggregation of type B* oligomers. (**a**) Particle morphology and interoligomer associations of type B* oligomers of WT and C-terminal deletion mutants of α-syn were determined by EM, scale bar 50 nm. (**b**) Type B* oligomers of the different variants were mixed with the human disaggregase, and their disassembly was analyzed by a sucrose-gradient fractionation (**c**). In the case of α-syn_WT_ and α-syn_1-122_, the same reactions were also analyzed with antibodies against each chaperone component of the human disaggregase.

It is now widely accepted that one of the most toxic species of α-syn are soluble oligomers generated during the early steps of the self-assembly process. In this context, we also wanted to know if the C-terminal truncation of such species could hamper the disaggregase activity of chaperones. To this aim, oligomers of the three C-terminal truncated mutants were obtained by lyophilization. These oligomers, previously termed type B* oligomers, are kinetically trapped during the self-assembly process [37,40,41]. Their spectroscopic and biochemical behavior are similar to those of the transient oligomeric and toxic forms generated during aggregation [42,43], despite being obtained under different conditions [44]. They offer the interesting possibility to explore whether chaperone-induced remodeling of these aggregation intermediates also depends on the presence of the C-terminal region of α-syn. The CD spectra of oligomers of the C-terminal truncated mutants showed the characteristic β-sheet minimum at 218 nm, with a loss of ellipticity that became especially evident upon deletion of the last 18 residues (Figure S4a). This behavior was similar to that described for fibrils and indicates that type B* oligomers of the shorter truncated mutants undergo a conformational rearrangement as compared with the WT protein (Figure 1d). Upon C-terminal truncation, type B* oligomers showed a gradual increase in the particle size, as seen by DLS (Figure S4b), suggesting an increased tendency to associate. EM confirmed DLS measurements and showed that, as the C-terminus shortens, more cylinder-like particles were able to associate (Figure 5a). 

Chaperone-mediated disaggregation of type B* oligomers of the four different protein variants was studied by a sucrose-gradient fractionation. As expected, in the absence of chaperones they appeared in intermediate fractions of the gradient (Figure 5b). Upon chaperone addition, α-syn_WT_ was fully disaggregated and moved as monomers to the top of the gradient. In the case of the truncated variants, the disaggregase activity was progressively reduced as the length of the deleted fragment increased. Interestingly, this reduction was accompanied by an increase in the amount of protein at the bottom of the gradient, which may indicate an increase in size upon chaperone binding. This was confirmed by comparing the fraction-distribution of chaperones in disaggregation mixtures of α-syn_WT_ and α-syn_1-122_, which showed an increased proportion of DnaJB1 and Hsc70 in the heaviest fractions for the truncated mutant (Figure 5c). Therefore, we conclude that C-truncation of α-syn favors the association of toxic oligomers, hampering their chaperone-induced disassembly without affecting their binding. 

## 3. Discussion

The Hsp70-based chaperone machinery constitutes a powerful ATP-dependent amyloid disaggregase that efficiently disassembles α-syn fibrils [19,20,21,45]. We show herein that α-syn fibrils lacking the last 30 C-terminal amino acids are not disassembled by this chaperone machinery, whereas those without the N-terminus are solubilized similarly to the WT fibrils by the chaperone mixture, in agreement with recently published data [21]. This behavior was initially explained considering that the C-terminal end of α-syn contains the binding sites for DnaJB1, whose initial interaction with the aggregate promotes productive Hsc70 recruiting to the fibril surface [19,21]. Deletion of the last 30 residues of α-syn increases the K_d_ values for DnaJB1 binding to fibrils from 700 to 4000 nM [21]. We observe this reduction in the affinity as a nearly twofold decrease in the amount of fibril-bound DnaJB1. Despite this difference, the cochaperone bound to α-syn_1-110_ fibrils recruits an amount of Hsc70 molecules similar to that estimated for α-syn_WT_. Additionally, we show that although α-syn_1-110_ can seed aggregation of α-syn_WT_, transferring its secondary structure, chaperones were able to disaggregate these cross-seeded fibrils. On the contrary, seeding of α-syn_1-110_ monomers with α-syn_WT_ aggregates resulted in fibrils with a WT-like conformation that challenged the disaggregase. Thus, the lack of chaperone-induced disaggregation of C-truncated fibrils is not due to a change in their secondary structure nor to a decrease in the number of Hsc70 molecules cooperating at the fibril surface.

**Figure 6:**
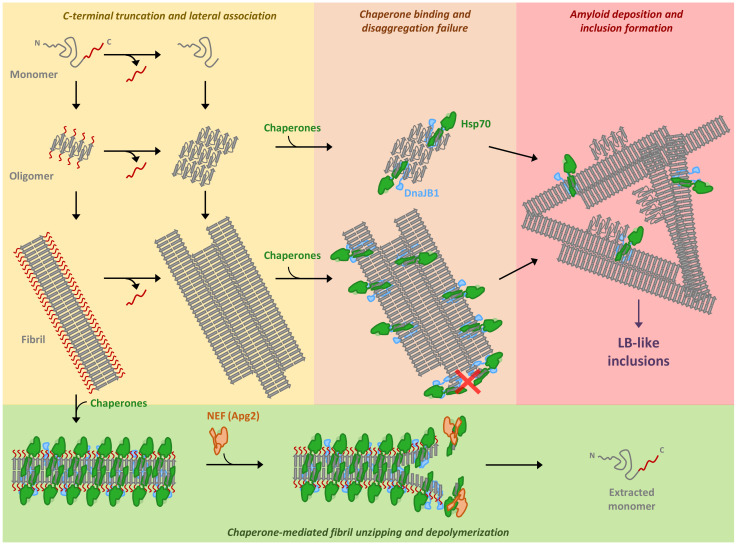
Emerging impairment of the human disaggregase activity due to C-terminal truncation of α-synuclein. α-Synuclein C-terminal truncation happens physiologically at different stages of the aggregation process. Here, we show that truncation of both oligomers and fibrils results in their lateral association. Although chaperones can bind to laterally associated aggregates, their disaggregation activity is blocked. We propose that lateral association could impair the chaperone-mediated protofilament unzipping required for fibril disassembly. This loss of chaperone activity may be linked to the enhanced propensity of C-truncated species to deposit in vivo, which seems to be a mayor regulator in the formation of Lewy body-like inclusions.

α-Syn C-terminal truncation enhances aggregation in vitro [16,17,46,47] and in vivo [48,49,50]. The protective role proposed for the charges at the C-terminus via long-range intramolecular interactions is lost upon truncation, which favors aggregation [18,51,52,53,54]. C-terminal charges also seem to play an important role after fibril formation, generating an interfibrillar electrostatic repulsion. Deletion of the C-terminal region abrogates these long-range repulsive interactions, promoting lateral interfibrillar associations into higher-organized suprafibrillar aggregates [32,33,38,39]. We show herein that such aggregates pose a challenge for the human disaggregase. This is evidenced with the recombinantly produced C-truncated mutants, which show a gradual reduction in fibril disassembly as the length of the deleted region at the C-terminus increases, correlating with the progressive increase in fibril lateral association described here and elsewhere [39]. Additionally, cleavage of α-syn_WT_ fibrils with calpain-1, one of the many proteases that target the α-syn C-terminus [10], also results in an increased lateral association accompanied by a reduction of the disaggregase activity of the chaperone mixture. This loss of chaperone activity can be explained taking into account the proposed mechanism for fibril disassembly [20,21]. In this model, DnaJB1 recruits Hsc70 molecules in a crowded state, binding up to every other α-syn protomer within the fibril [21]. Upon nucleotide exchange by Apg2, disassembly starts with the destabilization of the fibril ends and rapidly progresses to completion through protofilament unzipping and depolymerization [20] (Figure 6). We rationalize that the enhanced tendency of C-truncated fibrils to laterally associate into stacked assemblies could restrict the initial protofilament unzipping, arresting disaggregation (Figure 6). Additionally, the formation of fibrillar structures with a higher twist and a tighter packed core that results in an increased proteinase K resistance [25], in agreement with the higher stability found here (Figure S1c), might also impose a challenge for disassembly. We also show that this disassembly blockade due to truncation-driven lateral association extends to α-syn type B* oligomers (Figure 6). Interestingly, chaperone binding to C-truncated oligomers resulted in the formation of larger species, a similar behavior observed for other chaperone–oligomer complexes [55,56,57]. The human disaggregase targets toxic α-syn intermediates, including oligomers and short fibrils, and fully disassembles them to monomers in an all-or-none process [20]. Through this mechanism, the Hsp70-based machinery could function as a first barrier against amyloid formation, maintaining α-syn in its native and functional state. Our results suggest that C-terminal cleavage at early stages of amyloid formation greatly impacts the housekeeping function of the disaggregase. With a futile chaperone disassembling activity, C-truncated amyloid intermediates can accumulate, being able to exert their inherent toxicity and grow into larger aggregates that tend to deposit (Figure 6). Although deposition could seem beneficial in terms of toxicity neutralization, the formation of LB-like inclusions has also been suggested to be a major driver of neurodegeneration by disrupting cellular functions such as organellar trafficking and inducing mitochondria damage and deficits, all contributing to synaptic dysfunctions [58,59]. In this context, C-terminal truncation is a master regulator of α-syn inclusion formation [39,59], favoring formation of fibrillated protein scaffolds that sequester cytoskeletal elements, membrane fragments, vesicles, lysosomes, mitochondria and other misshapen organelles [58,60]. This is further evidenced by the fact that truncated variants of α-syn can account for up to 15–25% of the total α-syn, being enriched in Lewy-body-insoluble fractions in α-synucleinopathies and increasing their insolubility with the length of the truncated C-terminal region [8]. Furthermore, different forms of α-syn in nigral LBs and LNs show an onion-skin-like architecture, with a structured framework of α-syn phosphorylated at Ser129 and neurofilaments, encapsulating a core of C-terminally truncated α-syn [61,62]. Within this core, α-syn species with a larger deletion at the C-terminus tend to be located in the center [61]. These data correlate nicely with the gradual loss of chaperone activity upon deletion of C-terminal residues shown herein and suggest that the role of C-truncated species in LB-like inclusion formation may be linked to their ability to bypass the disaggregase action.

In summary, we provide here a molecular explanation for the elusive ability of the human disaggregase machinery to clear C-truncated α-syn amyloids. Loss of the inter-fibril electrostatic repulsion provided by the negative charges of the C-terminus allows them to laterally associate, resulting in an ordered, stacked structure that could be detrimental for fibril unzipping, a step necessary for fibril solubilization.

## 4. Materials and Methods

### 4.1. Protein Cloning, Expression, Purification and Labeling

Chaperones were produced as previously reported [30]. α-Syn deletion mutants were obtained by PCR amplification using α-syn_WT_ plasmid as template and cloned into an empty pT7-7 plasmid, except for α-syn_1-110_, which was obtained by site-directed mutagenesis. α-Syn_WT_, α-syn_Q24C_, α-syn_1-133_, α-syn1_-122_ and α-syn_30-140_ (vector pT7-7) were expressed and purified following a previously reported protocol [63]. α-Syn_1-122_ was further purified by Superdex 200 gel filtration in a buffer containing 25 mM Tris-HCl pH 7.5. α-Syn_1-110_ was expressed and purified as for α-syn_WT_, although using a HiTrap SP HP cation exchange column in 20 mM MES-NaOH pH 6.8, and elution was performed with a 0–0.6 mM NaCl gradient. Protein concentration was estimated from absorbance at 280 nm with the extinction coefficient of 5960 M^−1^cm^−1^ for α-syn_WT_ and α-syn_30-140_, 4470 M^−1^cm^−1^ for α-syn_1-133_ and 1490 M^−1^cm^−1^ for α-syn_1-122_ and α-syn_1-110_. α-Syn_Q24C_-Alexa Fluor™ 488 was obtained by following the same protocol previously described to label cysteine-containing variants with maleimide-derivatized fluorophores [44].

### 4.2. Aggregate Preparation

α-Syn oligomeric samples were prepared by lyophilization as previously described [41], obtaining an oligomerization efficiency of 3–5% for all α-syn variants except for α-syn_1-110_, which was lower (1–3%). Fibrils were prepared by incubating a solution of 100 μM α-syn at 37 °C under orbital agitation (1000 rpm) for 7 days in aggregation buffer (50 mM Tris, 100 mM NaCl and 0.05% NaN_3_, pH 7.4). For cross-seeding experiments, samples were first passed through 100 kDa molecular weight cut-off filters to remove any pre-existing aggregation nuclei. Then, 100 μM of purified α-syn monomers were seeded with 5% (mol/mol) preformed fibrils and incubated at 37 °C under quiescent conditions for 3 days. After incubation, fibrils were purified by centrifugation for 30 min at 16,000 g and 4 °C. Pelleted fibrils were resuspended in disaggregation buffer (40 mM Hepes pH 7.6, 50 mM KCl, 5 mM MgCl_2_, 2 mM DTT) and the final fibril concentration was determined by dissolving an aliquot of the preparation in 4M GnHCl and measuring its absorption at 280 nm. For disaggregation experiments, sonicated fibrils were obtained using a Branson 450 Digital Sonifier equipped with a tapered microtip of 3 mm diameter at 10% power, with a total of 90 sonication cycles of 1 s ON/1 s OFF, with the sample set on ice-cold water. This sonication step was introduced considering previously reported data showing that chaperone-mediated disassembly of α-syn_WT_ fibrils was more efficient for shorter fibrils (less than 20% disassembly at 2.5 h for unsonicated fibrils and more than 80% for sonicated ones) [20].

### 4.3. Sucrose Gradient Fractionation

α-Syn disaggregation was carried out in disaggregation buffer at final protein concentrations of 10 µM α-syn, 10 µM Hsc70, 5 µM DnaJB1 and 1 µM Apg2 in a total volume of 400 µL in the presence of ATP (2 mM) and an ATP-regeneration system (8 mM phosphoenol pyruvate and 20 ng/µL pyruvate kinase). After incubation (2.5 h at 30 °C), the reaction mixture was applied to 3.2 mL of a 5–40 % sucrose gradient. In the case of α-syn oligomers, sample and gradient volumes were halved to save protein. Samples were centrifuged at 162,000 g for 2 h at 4 °C and 400 µL fractions (200 µL for oligomers) were manually removed and subjected to SDS-PAGE and immunoblotting using an anti-α-syn antibody (Invitrogen PA5-85343, 1:2000 dilution). Alternatively, samples were analyzed using antibodies against Hsc70 (Abcam ab51052; 1:5000), Apg2 (Abcam ab185962; 1:5000) and DnaJB1 (Enzo ADI-SPA-450, 1:2000).

### 4.4. Disaggregation Kinetics

Disaggregation was carried out in 96 well half area black plates (non-binding surface; Corning^®^) at 2 μM α-syn sonicated fibrils (10–20% labeled with Alexa488) and the chaperone concentrations stated in each experiment. Before starting the reaction, samples were stabilized for 30 min at 30 °C in the plate reader. The reaction was initiated with the addition of ATP (2 mM) and an ATP regeneration system (8 mM phosphoenol pyruvate and 20 ng/µL pyruvate kinase), and afterwards plates were sealed with HD Clear Duck tape and measured. Fluorescence readings were collected every 3 min from the top using excitation and emission filters of 485/20 and 528/20 nm and a gain of 60–75. No disaggregation (0 %) and complete (100 %) disaggregation controls were obtained with aggregates alone or monomers of each protein variants in the presence of chaperones, respectively.

### 4.5. Aggregation Kinetics Measurements

Fibrillar aggregation of α-syn in the absence or presence of Hsps was monitored using a ThT fluorescence assay. α-Syn was incubated at 50 µM in aggregation buffer with 50 µM ThT at 37 °C under quiescent conditions with 5% (mol/mol) seeds (sonicated fibrils). Chaperones were added individually at 1:10 (chap/syn) molar ratio. The ThT fluorescence was measured in a Synergy HTX plate reader using excitation and emission filters of 400/30 nm and 485/20 nm, respectively, and readings were taken every 5 min for a period of up to 80 h.

### 4.6. Co-Sedimentation Assay

Unsonicated α-syn fibrils were diluted to 2 μM in disaggregation buffer containing Hsc70 (2 μM), DnaJB1 (1 μM) and Apg2 (0.2 μM). After adding 2 mM ATP, samples were incubated for 10 min and subsequently centrifuged at 16,000 g at 4 °C for 30 min. Proteins in pellets were analyzed and quantified by SDS-PAGE and densitometry.

### 4.7. Negative Stain Electron Microscopy

For electron microscopy, 3 μL aliquots (1–2 mg/mL) of the different samples were applied onto glow-discharged formvar/carbon-coated 200-mesh copper grids and incubated for 1 min. Grids were negatively stained with 1% (*w*/*v*) uranyl acetate (2 staining steps of 10 s) and air-dried for 5 min. Images of the fibrils were taken in a JEOL JEM 1400 Plus electron microscope. Images of type B* oligomers (α-syn_WT_, α-syn_1-133_, α-syn_1-122_ and α-syn_1-110_) were taken using a JEOL 1010 JEM electron microscope operated at 100 kV and equipped with a CCD camera (4 K × 4 K TemCam-F416, TVIPS). Images were recorded at a 65,000× nominal magnification with a pixel size of 15.50 μm (2.4 Å/px sampling rate). These images were processed following the Scipion2 processing workflow [64]. Images were CTF-corrected using CTFFIND4 [65]. Particles were automatically selected using Xmipp3 [66] and 2D-classified using Relion2 [67] and CryoSPARC (v2.14.2) [68]. The images shown in Figure 5 correspond to one of the larger and representative classes of each type B* oligomers obtained after 2D classification.

### 4.8. Dynamic Light Scattering

Size volume distribution profiles of the different α-syn samples (10 μM in disaggregation buffer) were obtained at 25 °C using a Zetasizer Nano ZS (Malvern Instruments) at a back scattering angle of 173°.

### 4.9. Circular Dichroism

Far-UV CD spectra of samples containing 10 μM α-syn in PBS were acquired at 20 °C in a Jasco J-810 circular dichroism spectropolarimeter using rectangular quartz cuvettes with 1 mm path length. Each spectrum represents the average of 15–20 scans, collected from 200–260 nm, with a spectral bandwidth of 1 nm and a response time of 1 s. 

### 4.10. FT-IR Spectroscopy

Aggregates (1–10 mg/mL) were exchanged into deuterated PBS buffer and applied on a 25 μm carved calcium fluoride window mounted in a Peltier cell (TempCon, Bio Tools). Spectra were collected with a nominal resolution of 2 cm^−1^ in a Nicolet Nexus 5700 spectrometer equipped with a MCT detector at 20 °C. They were digitally subtracted using a spectrum of deuterated buffer as reference, and the area of the amide I band (1700–1600 cm^−1^) was normalized in all spectra. 

### 4.11. Calpain-1 Digestion

Calpain-1 (Merck, 208712) cleavage of α-syn was carried out using previously described methods with some modifications [13]. Briefly, different amounts of calpain-1 (stated in each experiment) were added to a solution of 50 µM α-syn in buffer 40 mM Hepes pH 7.6 and 5 mM DTT. Reactions were initiated by the addition of CaCl_2_ (1 mM final concentration) and incubated at 37 °C for 10 min. Reactions were stopped by adding SDS-PAGE loading buffer and boiling for 10 min. To determine the effect of calpain cleavage on the secondary structure of fibrils, unsonicated fibrils were digested with 13.5 ng/µL of calpain-1, and after 10 min incubation at 37°C they were centrifuged (30 min at 17,000 g and 4 °C) and resuspended in PBS buffer. For EM experiments, after digestion (13.5 ng/µL of calpain-1) of unsonicated and sonicated fibrils, samples were incubated overnight with 12.5 µM of the calpain inhibitor VI (SJA 6017) (Santa Cruz Biotechnology, Santa Cruz, CA, USA). When sonicated fibrils were digested and used in disaggregation experiments, they were incubated for 2 h with 12.5 µM calpain inhibitor VI prior to their addition to the chaperone mixture. This inhibitor did not affect the disaggregase activity of the chaperones under the conditions used (see trace “0” in Figure 4 e).

## Supplementary Materials

The following are available online at https://www.mdpi.com/article/10.3390/ijms222312983/s1.
