# Supplementary material for: Truncation-Driven Lateral Association of α-Synuclein Hinders Amyloid Clearance by the Hsp70-Based Disaggregase
Source: Int J Mol Sci. 2021 Nov 30;22(23):12983. doi: 10.3390/ijms222312983 (PMC8657883; doi:10.3390/ijms222312983)
Supplement: Supplementary file 1 [file ijms-22-12983-s001.zip › ijms-1494338-supplementary.pdf]

Supporting Information for

Truncation-driven lateral association of α-synuclein hinders amyloid clearance by the Hsp70-based disaggregase

Aitor Franco, Jorge Cuellar, José Ángel Fernández-Higuero, Igor de la Arada, Natalia Orozco, José M. Valpuesta, Adelina Prado and Arturo Muga

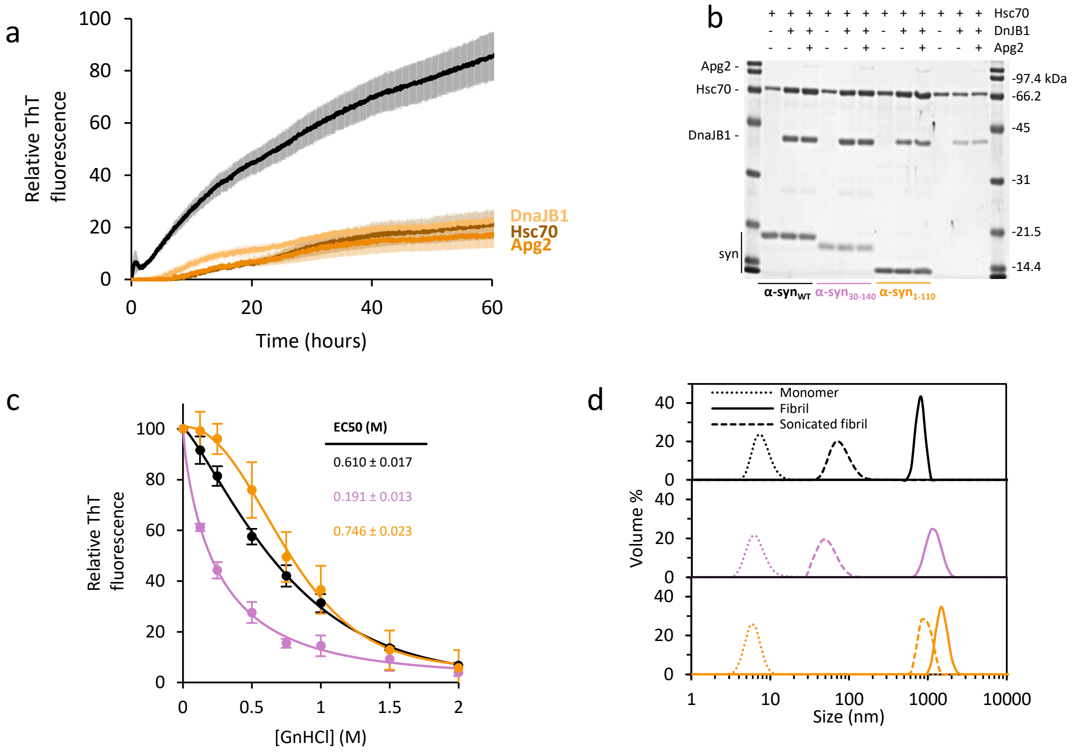

Figure S1. Chaperones interact similarly with WT and N- or C-terminally truncated α-syn fibrils. (a) Aggregation of self-seeded α-syn$_{1-110}$ followed by the increase in ThT fluorescence in the absence and presence of each of the three components of the human disaggregase system at 10:1 (α-syn:chaperone) molar ratio. Relative ThT fluorescence was obtained by subtracting the initial fluorescence of each sample. **(b)** Co-sedimentation of 2 µM α-syn fibrils (full-length or truncation mutants) and Hsc70 (2 µM) in the presence of DnaJB1 (1 µM) alone or in combination with Apg2 (0.2 µM). As controls, the same chaperone mixtures were analyzed in the absence of fibrils. The stability of the fibrils obtained with each variant was analyzed by GdnHCl chemical denaturation **(c),** and fragmentation susceptibility upon sonication as seen by DLS **(d)**.

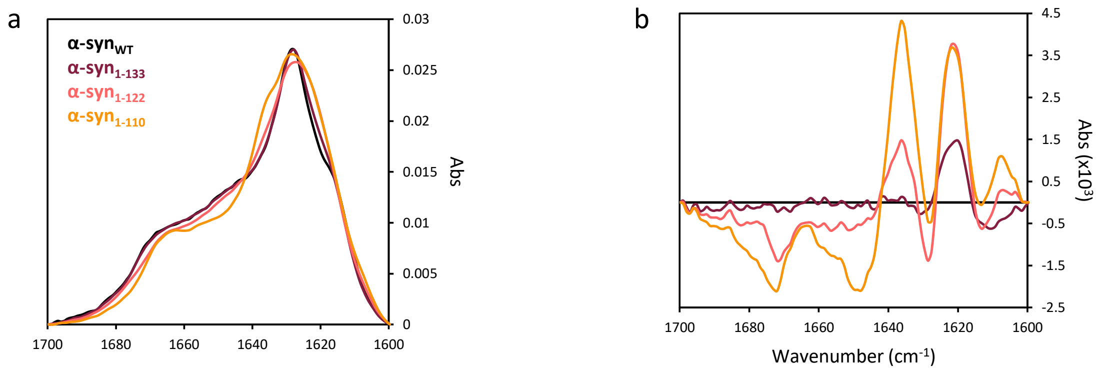

Figure S2. Structural analysis of C-truncated fibrils. Original **(a)** and differential **(b)** FT-IR spectra of α-syn variants. The differential ones were obtained after subtracting the spectrum of each variant from that of α-syn$_{WT}$.

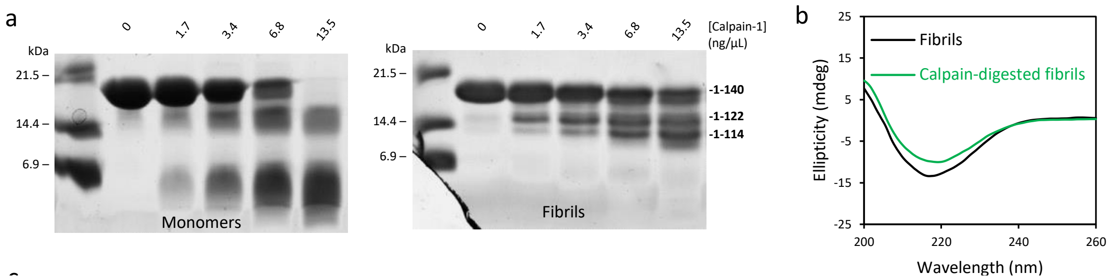

Figure S3. Calpain-1 digestion of α-synuclein. (a) Monomeric (left panel) or fibrillar (right panel) α-syn was incubated at 37°C for 10 min in the absence or presence of different calpain-1 concentrations and the samples were analyzed by SDS-PAGE. **(b)** Far-UV CD spectra of undigested and calpain-digested α-syn fibrils.

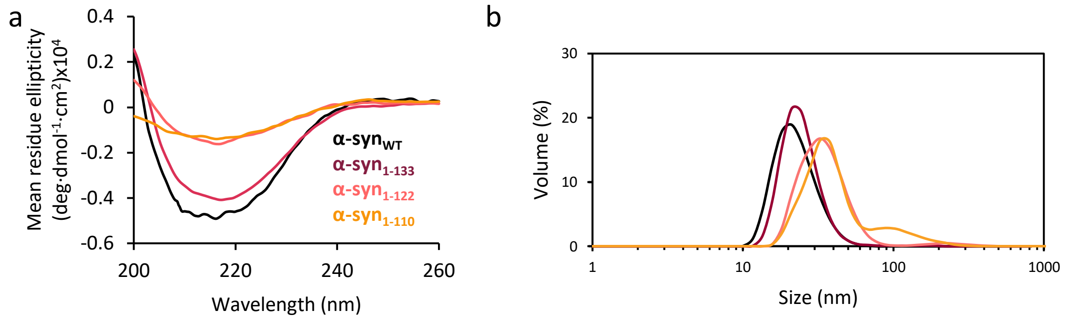

Figure S4. Structural and size characterization of C-truncated α-synuclein oligomers. Far-UV CD spectra **(a)** and DLS-derived size distribution **(b)** of type B oligomers of WT and C-terminal deletion mutants of α-syn.
